# Satisfaction With Services Among Attendees of Physiotherapy Outpatient Clinics in Tertiary Hospitals in Lagos State

**DOI:** 10.1177/2374373519847370

**Published:** 2019-05-24

**Authors:** Ijeoma Jane Odumodu, Tolulope Florence Olufunlayo, Babatunde Enitan Ogunnowo, Michael Ebe Kalu

**Affiliations:** 1 Nigerian Securities, Printing and Minting Staff Clinic, Lagos, Nigeria; 2 Department of Community Health & Primary Care, College of Medicine, 70670 University of Lagos, Lagos, Nigeria; 3 School of Rehabilitation Science, 62703 McMaster University, Hamilton, Ontario, Canada

**Keywords:** patient, satisfaction, physiotherapy services, tertiary hospitals, Lagos state

## Abstract

**Aim:**

To determine outpatients’ satisfaction with physiotherapy services in tertiary hospitals in Lagos State, Nigeria.

**Method:**

This cross-sectional study utilized a simple random sampling method to recruit 284 participants. Participants responded to a 2-part structured questionnaire with 33-items on a 5-point Likert-type scale. Participants were asked questions about their satisfaction with physiotherapy services, staff attitudes, and the accessibility of outpatient physiotherapy clinics. Data were analyzed using descriptive analysis and χ^2^.

**Results:**

About 28.8% of the participants were satisfied or very satisfied with the location of the outpatient physiotherapy clinics. The majority of the participants were satisfied or very satisfied with these physiotherapy services in maintaining privacy (86.2%), scheduling convenient clinic appointments (78.2%), prompt scheduling of the first physiotherapy clinic appointment (74.6%) and subsequent visits (78.9%), and providing a calm/relaxing atmosphere (90.1%). While 22.5% of the participants were satisfied or very satisfied with the waiting time in these physiotherapy clinics, 86.3% and 97.9% were satisfied or very satisfied with staff attitudes and the cost of the therapy, respectively. Almost all (97.9%) the participants reported being satisfied or very satisfied with their overall experience in the physiotherapy clinics. While there was an association between marital and educational status with overall satisfaction scores, age and gender showed no association.

**Conclusion:**

Our findings suggest that patients attending the outpatient physiotherapy clinics in tertiary hospitals in Lagos State, Nigeria, were satisfied or very satisfied with the domain that measured overall satisfaction. Strategies to reduce the waiting time in these physiotherapy clinics and to enhance physical accessibility of the physiotherapy clinics are encouraged.

## Introduction

The need for continuous improvement of quality and safety in the provision of patient care has become self-evident (1). The resultant paradigm shift from an acceptance of the status quo to a drive for constant improvement in clinical practice has required the engagement of multiple monitoring strategies (1). Ascertaining patients and their relatives’ satisfaction to care received is one of the most reliable strategies to improve clinical practice (1).

Patient satisfaction refers to the extent to which the patients perceive that their needs and expectations are met by the services provided (1). It is often related to health outcomes that are consistent with the patient’s values and preferences (2). Patients rate their satisfaction using different constructs including the art of care (caring attitude), technical quality of care, continuity of care, accessibility and convenience, finances (ability to pay for services), physical environment, efficacy, and outcome of care (3). Individual patient satisfaction reports may be mediated by variables such as age, reported health status, ethnicity, gender, engagement with the system, faith and gratitude, or perceptions of what constitutes a “good” health-care professionals (4). These variables have been demonstrated to predict patient satisfaction scores (4). Research has suggested some other factors that could influence patient satisfaction across all care settings; such factors include respect for patient preferences, emotional support, involvement of family and friends, continuity and transition, physical comfort, empathy, and personalized therapy (5). Understanding some of these features have informed the care approach in the Western world (6,7). For instance, the patient-centered approach to care was informed by evidence of studies that explore patient satisfaction (8,9). Patient satisfaction is related to the best achievable health outcome such as improved quality of life and reduced health-care cost (10–13). While health-care services in developed countries have utilized the health outcomes associated with patient satisfaction (14,15) the use of patient satisfaction to inform care practices are evolving in developing nations such as Nigeria (16,17).

In Nigeria, the attitude of health workers, long waiting times, cost of care, hospital bureaucracy, and easy access to alternative medical practices are serious barriers to the uptake of orthodox medical services (2). Patients who are satisfied with the quality of care are likely to seek medical consultation in the hospital, adhere to a treatment plan, maintain a continuous relationship with the hospital, recommend the hospital to others in the community, make informed choices about the health-care providers, and encourage a continuous quality improvement in the hospital (2).

Throughout the world, musculoskeletal health problems are major morbidity issues for indigenous populations, and the social and economic burdens imposed by musculoskeletal complaints are significant (18). Concomitant with the increasing aging population is also a significant increase in the prevalence of chronic diseases; these have increased the need for physiotherapists and physiotherapy services by all health agencies across the globe (19). Physiotherapists’ expertise in movement and exercise and their in-depth knowledge of the pathophysiology of acute and chronic diseases and injuries make them an obvious choice to address the health-care needs of the adult population (20). According to the World Confederation for Physical Therapy in 2018, physiotherapy workforce has a key role to play in the public health agenda through its contribution to the prevention of disease, promotion of good health, particularly through physical activity and improvement in the general quality of life (21). Physiotherapy has several characteristics that may influence patient satisfaction. Physiotherapy treatment involves physical contact; therapy usually requires the patient’s active participation in a long length of interaction with physiotherapists (22).

The tertiary hospitals in Nigeria operate in societies made up of people with various sociodemographic backgrounds. Lagos, for instance, being the most economically important state in Nigeria (23) and currently the most populated region in Nigeria (about 21 million people as of 2016), (24,25) is a sociocultural melting pot attracting both Nigerians and foreigners alike (26). These tertiary institutions are often seen as authorities in several issues relating to health, thereby creating high expectations with regard to quality of care (2) However, in recent times, government-owned tertiary hospitals have received a lot of negative comments ranging from poor quality of service delivery to service delays, discontinued service, staff attitudes of indifference, and rushed bureaucratic procedures (27) This has led to poor public confidence in health care and made these hospitals unattractive to the consumers of health services (28).

Market expectations of physiotherapists reflect changing demands of health-care for client-centered management of conditions in an aging population (29). Despite the fact that there is a large body of literature supporting the importance of physiotherapy for optimal public health of every nation’s citizenry, there is still a need for a more public health-oriented evidence-based physiotherapy practice that meets the growing need of the Nigerian population (19). Also, there has been a preponderance of literature on patient satisfaction with other medical services, but little has been done with regard to physiotherapy (30). This study aimed at determining satisfaction with services in patients attending the outpatient physiotherapy clinics in tertiary hospitals in Lagos state, Nigeria. This study is one of a kind as it was done in the 2 teaching hospitals in Lagos State; while one is managed by the state government, the other is established and managed by the federal government. Findings from this study identified the gaps that exist in the delivery of quality physiotherapy services to patients in one of the most populated cities in Africa and the projected fifth largest economy in Africa by 2020 (26).

## Methods

### Study Setting

This study was conducted in the physiotherapy department of 2 teaching hospitals located in Lagos State, Nigeria. These 2 hospitals are quite similar with the same number of patient inflow and units in the physiotherapy department. Each of the physiotherapy departments in these hospitals is divided into different units including neurology, orthopedics, obstetrics and gynecology, general health promotion, and pediatric units, and each of these units has outpatient and inpatient sections. However, participants were recruited from outpatient clinics of the neurology, orthopedics, general health promotion (eg, patients living with obesity or human immunodeficiency virus, multiple chronic conditions—diabetes and hypertension), and obstetrics and gynecology. On a daily basis, an average number of 30 to 50 patients attend the outpatient units of the selected physiotherapy departments.

### Study Design and Participants

The study was a descriptive cross-sectional study; prospective participants were patients attending the adult outpatient clinics of the 2 teaching hospitals in Lagos State, Nigeria. Inclusion criteria for participants were that they (1) had attended at least 4 treatment sessions in the clinic (31); (2) must be at least 18 years old; and (3) must be able to communicate fluently in either English or Nigerian Pidgin.

### Sampling Method

A simple random sampling was employed to recruit the participants. A sample size of 398 participants was estimated using the 79% estimated proportion of patients satisfied with physiotherapy service and making allowance for 10% nonresponse rate based on previous studies (31). To ensure that participants selected reflected the representation of the 4 units of the 2 outpatient physiotherapy clinics, an estimate of 50 patients from each of the 4 units of the 2 outpatient physiotherapy clinic was made. Patients were randomly selected on a daily basis. The medical record officer provided the list of all patients that attend each clinic daily. Patients on the list with even numbers were approached by research assistants. If the patients met the inclusion criteria, the patient was asked to participate in the study. Recruitments stopped at any clinic that reached a maximum of 50 participants.

#### Study instrument

Participants were surveyed using an adapted version of the Physical Therapy Patient Satisfaction Questionnaire (PTPSQ) developed by Goldstein et al (32). The original questionnaire is a valid and reliable tool for assessing patient satisfaction for physical therapy services (32). Since the original tool was developed to test patient satisfaction of the physical therapy services and care in the United States, modifications of the PTPSQ to reflect the cultural context was performed. In this section, physical therapy, as opposed to physiotherapy (both meaning the same thing) was used in order to maintain the originality of the tool adapted in this study.

#### Instrument content

A panel of 10 experienced patients with at least 1 year experience of attending physiotherapy outpatient clinic, 2 clinicians, and 3 researchers with at least 5 years’ of experience in questionnaire development were invited for face and content validity. All the members of the panel received the original Physical Therapy Patient Satisfaction Questionnaire (PTSPQ), (32) and they were asked to revise and/or add question(s) specific to the physiotherapy practice context in Nigeria. Revisions to questions wording and answer options were made following each of 2 rounds of reviews. For the demographics, questions such as ethnicity, marital status, educational status, occupations, insurance type, and outpatient clinic visited on the day of interviewing were added. For the questions that assessed patient satisfaction for physical therapy services, the question “ I was satisfied with the services provided by my physical therapy assistant(s)” was removed because most physiotherapy clinics in Nigeria do not use the services of physical therapy assistants. Two questions (my bills were accurate and if I had to, I would pay for these physical therapy services myself) that asked about finances were removed because the questions were reductant, since more than 90% of the patients who use physical therapy services in Nigeria pay out of pocket (1) Also, physical therapy was changed to physiotherapy, which is the term patients are familiar with in Nigeria context. Five (questions 3,4, 5, 11 and 15) questions were added to the questionnaire to reflect the physiotherapy practice context in Nigeria (See Appendix A). The resulting modified questionnaire was further subject to some psychometric properties testing including internal consistency (reliability), concurrent validity, and exploratory factor analysis (structural validity). The detailed plan of the psychometric test plan is explained in the analysis section. At the end, the modified version of Goldstein et al (32) PTPSQ contains 33 questions with 2 sections—10 questions on demographics and 23 questions asked about the patient’s satisfaction on a 5-point Likert-type scale that ranges from “strongly disagree” to “strongly agree.” To ensure confidentiality and no influence of the therapists, the patients were asked to fill the questionnaire at the point of exit and were instructed to drop the questionnaire in a designated box provided. Data were collected for a period of 3 months.

### Data Analysis

Data entry and analysis were done using Software Statistical Package for Social Scientists (SPSS) version 13.0. Descriptive statistics were calculated for all the sociodemographic characteristics. The items that measure satisfaction were presented in frequencies and percentages. χ^2^ was used to determine the statistical association between variables. All tests were conducted using a 95% confidence level unless otherwise stated. For reliability, the internal consistency (item by total) of the questionnaire was performed using a 2-way random effect to investigate where items correlated significantly with each other, and the findings were interpreted based on Koo and Mae guidelines (33). The test–retest reliability was not performed because it is possible that this test would not give a reliable measure of the questionnaire over time, since the participants may experience “selective forgetting” causing them to perceive physiotherapy care differently as time elapses (32). Concurrent validity was performed using the method employed by the originators of the questionnaire (32). In summary, the originators of the questionnaire used the overall satisfaction questions consisting of the following: would recommend physical therapy service to family and friends; would return to the facility for physical therapy in future; and overall satisfaction with physical therapy experience as their criterion variable. These questions correspond to the questions 21, 22, and 23 in the modified PTPSQ questionnaire. The summary scores of other variables were correlated with each of the 2 criterion variables (32,34). An exploratory factor analysis of the 23 items in the PTPSQ was performed to evaluate the factor structure relative to the 4 dimensions of patient satisfaction described by Nelson (35): access, administrative technical management, clinical technical management, interpersonal management, and continuity of care.

### Ethical Considerations

Ethical approval for the study was obtained from the Health-Research and Ethics Committees (H-REC) of the Lagos University Teaching Hospital (LUTH) and Lagos State University Teaching Hospital (LASUTH). Informed consent was obtained from the participants before the study, and they were assured of confidentiality for information supplied.

## Results

Of the 398 selected to participate in this survey, only 284 completed the questionnaire, giving a response rate of 71% of which 52%, 25%, 13%, and 10% were from orthopedic, general health promotion, neurology, and obstetrics and gynecology, respectively. Participants have been attending the outpatient physiotherapy clinic for between 6 months and 10 years, with a mean of 4 years (SD = ±1.46). The majority (81%) of the participants were employed. Almost half (48%) of all participants had attained to secondary level of education. Almost all the participants pay out of pocket for health care (98.6%), while the remaining were insurance payee. Other demographic information is in given in Table 1.

**Table 1.** Demographic Characteristics and Their Association With Their Overall Satisfaction With Physiotherapy Service.

Variable	Total	Satisfied	Not Satisfied	Chi-Square
	F (%)	F (%)	F (%)	
Age (mean age = 50.75±14.97				
≤30	32 (11.3)	32 (100.0)	0 (-)	χ^2^ = 4.114
31-40	58 (20.4)	57 (98.3)	1 (1.7)	df = 4
41-50	48 (16.9)	48 (100.0)	0 (-)	*P* = .091^a^
51-60	61 (21.5)	58 (95.1)	3 (4.9)	
>60	85 (29.9)	83 (97.6)	2 (2.4)	
Gender				
Male	87 (30.6)	84 (96.6)	3 (3.4)	χ^2^ = 1.082
Female	197 (69.4)	194 (98.5)	3 (1.5)	df = 1
				*P* = .375^a^
Marital status				
Single	34 (12.0)	33 (97.1)	1 (2.9)	χ^2^ = 7.231
Married	228 (80.2)	224 (98.2)	4 (1.8)	df = 3
Others (divorced and widowed)	22 (7.8)	21 (95.5)	1 (4.5)	*P* = .046^b^
Educational status				
No formal Education	1 (-)	1 (100.0)	0 (-)	χ^2^ = 10.630
Primary Education	54 (19.4)	54 (100.0)	0 (-)	df = 5
Secondary Education	134 (48.0)	131 (97.8)	3 (2.2)	*P* = .017^b^
National Diploma	40 (14.0)	39 (97.5)	1 (2.5)	
Undergraduate degree	34 (13.0)	33 (94.1)	1 (5.9)	
Postgraduate degree	15 (5.0)	14 (93.3)	1 (6.7)	
Ethnicity				
Yoruba	119 (42.0)	116 (97.5)	3 (2.5)	
Igbo	148 (52.1)	142 (96.0)	6 (4.0)	χ^2^ = 6.598
Hausa	13 (4.5)	13 (100.0)	0 (-)	df = 3
Others	4 (1.4)	4 (100.0)	0 (-)	*P* = .450^a^
Condition				
Ankle sprain	24 (8.5)	24 (1000.0)	0 (-)	χ^2^ = 2.791
Low back pain	45 (16)	43 (96.0)	2 (4.0)	df = 13
Cervical pain	24 (8.5)	24 (100.0)	0 (-)	*P* = .246^a^
Osteoarthritis	20 (7.0)	19 (95.0)	1 (5.0)	
Osteoporosis	12 (4.2)	10 (83.3)	2 (16.6)	
Rheumatoid arthritis	8 (2.8)	8 (100.0)	0 (-)	
Hip/knee fracture	18 (6.3)	15 (83.3)	3 (16.6)	
Falls	7 (2.5)	7 (100)	0 (-)	
Diabetes	12 (4.2)	9 (75.0)	3 (25.0)	
Obesity	15 (5.2)	14 (93.3)	1 (16.7)	
Stroke	57 (20.07)	50 (87.7)	7 (12.3)	
Parkinson disease	14 (4.9)	12 (85.7)	2 (14.3)	
Pelvic pain	7 (2.5)	7 (100.0)	0 (-)	
Fecal/urinary incontinence	21 (7.2)	19 (90.4)	3 (9.6)	
PT clinic attendance				
≤4years	157 (55)	150 (95.5)	7 (4.5)	χ^2^ = 1.987
>4 years	127 (45)	124 (97.6)	3 (2.4)	df = 1
				*P* = .370^a^

Abbreviation: PT, physiotherapy.

^a^Fisher exact value.

^b^Statistically significant.

### Psychometric Characteristics of the Instrument

The Cronbach α coefficient is 0.89, which shows a high degree of consistency across the measures. Table 2 shows the item analysis results and the Cronbach α coefficients that would be generated if each item were to be deleted from the instrument. The obtained correlations were *r* = .78 (*P* < .001) for question 21, *r* = .83 (*P* < .001) for question 22, and *r* = .80 (*P* < .001) for question 23 and summary score of other questions (concurrent validity). An exploratory factor analysis of the 23 items in the PTPSQ was conducted. Using an oblique rotation and a principal axis method for extraction, the result yielded a 4-factor solution. The initial eigenvalues indicated that the first 4 factors explained 28%, 12%, 8%, and 7% of the variance, respectively. The fifth and sixth factors had eigenvalues just over 1 and explained 5% of the variance. Most of the questions were loaded in the first six factors; however, after rotation, all factors were loaded on the first 4 factors. Items with factors loading of .40 or higher were used to define a factor (36). Items were placed on the factors of which they have the highest loading. Factor 1 consists primarily of 5 items that measure accessibility to the physiotherapy department, while factor 2 is comprised mainly of 6 items that measure physiotherapy services satisfaction. Factor 3 is comprised primarily of 6 items, and factor 4 consists primarily of 6 items that measure overall satisfaction of physiotherapy services. All items in the Table 3 show the factor loadings and the communalities.

**Table 2.** Reliability Analysis With Each Question Deleted.

No of Deleted Question	Scale Mean	Corrected Item Total Correlation	Cronbach α
1	86.73	.7657	.8878
2	86.63	.7878	.8889
3	86.20	.7378	.8778
4	86.10	.7566	.8790
5	86.14	.7278	.8626
6	85.14	.8012	.8999
7	85.63	.8212	.9012
8	85.92	.7890	.8890
9	86.91	.7512	.8772
10	85.51	.7624	.8771
11	85.36	.8024	.8234
12	85.31	.8712	.9012
13	85.46	.7651	.8900
14	85.63	.6789	.9025
15	85.39	.8990	.8900
16	85.39	.7896	.8912
17	85.51	.8900	.8789
18	85.61	.7900	.8790
19	85.40	.8300	.8677
20	85.36	.7906	.9010
21	85.39	.8600	.8900
22	85.37	.8678	.8769
23	85.89	.8976	.8189

**Table 3.** Factors Loading and Communalities Based on a Principal Components Analysis With Oblimin Rotation for 23 Items of the Adapted Version of the Physical Therapy Patient Satisfaction Question (Goldstein et al^32^).

Variable	Physiotherapy Accessibility	Physiotherapy Service	Staff Attitude	Overall Satisfaction	Communalities
1	.75				.53
2	.56	.32			.44
3	.66				.36
4	.74		.33		.46
5	.62	.34			.40
6		.67	.45		.56
7		.69	.54		.52
8		.56	.33		.67
9		.67	.45		.66
10		.76	.34		.34
11		.67	.48		.55
12		.52	.64	.32	.59
13			.89		.66
14			.88		.56
15		.34	.77		.67
16			.76	.67	.33
17		.33	.67		.44
18				.77	.67
19		.45		.55	.33
20		.34	.45	.52	.49
21				.89	.65
22		.45		.76	.56
23		.23		.78	.67

### Satisfaction Within the 4 Domains

Table 4 shows the percentages of the participant level of satisfaction in the 4 domains (physiotherapy accessibility, physiotherapy service, staff attitude, and overall satisfaction) of the questionnaire, ignoring the percentages of those that reported being indifferent in each item of the 4 domains. While 44% of the participants were very dissatisfied or dissatisfied about the location of the physiotherapy clinic, 42.1%, 45%, 46.5%, and 50.7% reported being satisfied or very satisfied with the available parking spaces, distance of the physiotherapy facility from the parking spaces, the accessibility features of the physiotherapy clinic, and reporting process at the front desk, respectively. Majority of the participants were very satisfied or satisfied with the physiotherapy services in maintaining privacy when needed (86.2%), scheduling clinical appointment at a convenient time (78.2%), prompt scheduling of first physiotherapy visit (74.6%) and subsequent visits (78.9%), and providing calm/relaxing atmosphere (90.1%). While 39.4% of the participants were very dissatisfied or dissatisfied with the waiting time in the physiotherapy clinics, 22.5% were very satisfied or satisfied with the waiting time. A good percentage of patients were satisfied or highly satisfied with the attitude of nonclinical staff (88%) and physiotherapists (92%), the physiotherapists’ understanding of their condition (95.1%), physiotherapists’ eagerness to reassuring them of their condition (89.1%), physiotherapists’ explanation of the treatment procedure (92.9%), and clear instruction during treatment and home program (93.3%). A similar trend was seen in the overall satisfaction domain of the question (see Table 4).

**Table 4.** Respondents Level of Satisfaction Level in the 4 Domains Measured.

	Very Dissatisfied, Freq. (%)	Dissatisfied, Freq. (%)	Indifferent, Freq. (%)	Satisfied, Freq. (%)	Very Satisfied, Freq. (%)
Physiotherapy accessibility (n = 5)					
Location of the physiotherapy department	38 (13.4)	87 (30.7)	77 (27.1)	66 (23.2)	16 (5.6)
Distance to get to the facility	11 (3.9)	36 (12.7)	109 (38.4)	118 (41.5)	10 (3.5)
Available Parking space^a^ (n = 126)	19 (15.1	26 (20.6)	28 (22.2)	38 (30.2)	15 (11.9)
Accessibility of the physiotherapy clinic	1 (0.4)	38 (13.4)	113 (39.8)	119 (41.9)	13 (4.6)
Admission/Entry and Administrative procedure	2 (0.7)	32 (11.3)	106 (37.3)	125 (44.0)	19 (6.7)
Physiotherapy service (n = 6)					
Maintenance of privacy when needed	0 (0.0)	8 (2.8)	31 (10.9)	206 (72.5)	39 (13.7)
Schedule of clinical appointment at convenient time	0 (0.0)	9 (3.2)	53 (18.7)	197 (69.4)	25 (8.8)
Prompt schedule of first physiotherapy visit	1 (0.7)	9 (3.2)	61 (21.5)	192 (67.6)	20 (7.0)
Easy to schedule subsequent visit after the first appointment	1 (0.4)	10 (3.5)	49 (17.3)	203 (71.5)	21 (7.4)
Waiting time	27 (9.5)	82 (28.9)	111 (39.1)	56 (19.7)	8 (2.8)
Calm and relaxing atmosphere in physiotherapy rooms	0 (0.0)	3 (1.1)	25 (8.8)	109 (59.5)	87 (30.6)
Staff attitude (n = 6)					
The non-clinical staff were helpful and courteous	0 (0.0)	3 (1.1)	30 (10.6)	213 (75.0)	38 (13.4)
Courteous of the physiotherapist	0 (0.0)	0 (0.0)	23 (8.1)	165 (58.1)	96 (33.8)
Therapist understanding of problems or health condition.	1 (0.4)	2 (0.7)	11 (3.9)	167 (58.8)	103 (36.3)
Easiness and reassurance of physiotherapist	0 (0.0)	2 (0.7)	29 (10.2)	165 (58.1)	88 (31.0)
Physiotherapist’s explanation of treatment process	0 (0.0)	5 (1.8)	15 (5.3)	158 (55.6)	106 (37.3)
Physiotherapist’s instruction	1 (0.4)	4 (1.4)	14 (4.9)	163 (57.4)	102 (35.9)
Overall satisfaction (n = 6)					
Cost of therapy	2 (0.7)	8 (2.8)	22 (7.7)	155 (54.6)	97 (34.2)
Quality of care received compared to the cost	5 (1.8)	13 (4.6)	43 (15.1)	135 (47.5)	88 (31.0)
Quality of therapy received	2 (0.7)	1 (0.4)	12 (4.2)	168 (59.2)	101 (35.6)
Overall satisfaction with physiotherapy experience	0 (0.0)	2 (0.7)	4 (1.4)	179 (63.0)	99 (34.9)
Recommendation of the physiotherapy department to family and friends	0 (0.0)	2 (0.7)	12 (4.2)	168 (59.2)	102 (35.9)
Would return to the physiotherapy department again	0 (0.0)	4 (1.41)	11 (3.9)	165 (58.1)	104 (36.6)

^a^Not all participants have a car, so the question was reductant for them and majority of them did not answer the question.

χ^2^ test showed that gender, age, ethnicity, participants’ self-identified diagnosis for visiting the outpatient physiotherapy clinic and length of attendance at the physiotherapy clinic were not associated with overall satisfaction in the respondents. However, a significant association was found between marital status and overall satisfaction with physiotherapy service, χ^2^ (3) = 7.231; *P* = .046. There was a significant association between the educational status of the participants and the overall satisfaction with physiotherapy, χ^2^(5) = 10.630; *P* = .017. There was an association between satisfaction with cost of treatment and the overall satisfaction with physiotherapy service, χ^2^(4) = 28.037; *P* = .002. The waiting time satisfaction was not significantly associated with the overall satisfaction with physiotherapy services, χ^2^(4) = 0.626; *P* = .960.

## Discussion

The aim of the study was to determine the satisfaction with services in patients attending the outpatient physiotherapy clinics in tertiary hospitals in Lagos State, Nigeria. Generally, most participants were satisfied or very satisfied with the overall measures of satisfaction, with most of them reporting indifference about the location and accessibility of the physiotherapy clinic. This finding is consistent with the literature (27). Our study identified that almost all the participants were paying out of pocket for the physiotherapy services. This supports the widely documented literature that Nigeria still has a very high out-of-pocket expenditure in health and that utilization of the National Health Insurance Scheme in Nigeria is still a far cry from what is obtainable in other developing countries such as Ghana (2).

The structural validity and reliability of the assessment tool after modification is an added contribution to the literature, and our findings are partially consistent with the psychometric properties reported by the originators (32). While the range of correlation of items in the original questionnaire was 0.58 to 0.97, (32) the range obtained in this study after the modification of the questionnaire was 0.67 to 0.90. The possible explanation to this range variation could be the fact the item—“if I had to, I would pay for these physical therapy services myself”—which has the lowest correlation (0.58) with other items was removed in the modified version used in this study. Interestingly, all the interitem correlations in the modified version of the questionnaire are above 5, which means that the variables are reliable. While the originators of the PTPSQ were concerned that the questionnaire yielded 1 factor, (32) the factor analysis in this study yielded 4 factors. Although the factors identified in our study were not an “exact” replica of the 5 dimensions (access, administrative technical management, clinical technical management, interpersonal management and continuity of care) identified by Nelson (35), it is of note the participants in our study identified all the components of the 5 dimensions but was yielded under 4 factors. For instance, it is expected that item 5—it was easy admission and administrative procedure when attending physiotherapy clinic—to have load on administrative technical management dimension. However, the item 5 had a loading factor of 0.62 on factor 1 (physiotherapy accessibility). Nevertheless, the 4 factors that yielded in our study confirm the argument that patient satisfaction is a multidimensional phenomenon (32).

Most of the participants were indifferent across most of items that measures how accessible the outpatient physiotherapy clinics are. However, about 44.1% of the participants reported being very dissatisfied or dissatisfied with the location of the outpatient physiotherapy clinics. This level of dissatisfaction has been reported among patients visiting their family doctor; patients identified that the location of the family doctor’s clinic is one of the major causes of their dissatisfaction (37). This is disturbing because the characteristics of the patients who attend physiotherapy clinics are often living with disability; therefore, being able to access the outpatient physiotherapy clinics contributes extensively to their overall satisfaction of the physiotherapy services.

On staff attitude and service delivery, most participants were satisfied with all the determinants measured. It is not surprising that a greater percentage reported being either indifferent or dissatisfied with the waiting time in these clinics in our study. Aside that, this finding is consistent across studies that have reported patient’s satisfaction with waiting time (27,38,39). We also argue that the grossly inadequate physiotherapist–patient ratio in these hospitals would have contributed to the increased wait time. For instance, the patient/clinician ratio for physiotherapy in Nigeria is 0.047 per 1000 of the population (40). Currently, physiotherapy services have not been incorporated into primary health-care services; therefore, every patient who requires physiotherapy is referred to either the secondary or the tertiary care centers except for a few who can afford the private facilities (41). As a result of this over dependence on the physiotherapy service at the secondary and tertiary level, the patient caseload as well as the wait time is likely to continue across various clinics in Nigeria. We believe that teaching most of the patients the process of managing their health conditions using the self-care approach could be promising and will likely reduce the waiting time at the clinics.

Most of our participants were satisfied that physiotherapists understood their conditions, were eager to reassure them, explained treatment procedures, and provided clear instructions for home programs. There is a wide range of literature supporting patients satisfaction with the interpersonal and clinical skills of the physiotherapist (42–44). Also, patient satisfaction has been reported higher following treatments delivered by physiotherapists compared to general medical practitioners (45). This may be as a result of the longer contact time physiotherapists spend with their patients compared to general medical practitioners (22). This satisfaction level could be a pointer that self-management approach will thrive in the Nigerian context, if handled by professionals that have high level of satisfaction (eg, physiotherapists) from the patients. Further research, however, is needed to confirm this assertion.

Based on the general report given by the participants, a central issue for health providers is finding out what patients do as a result of their satisfaction with care (43). Do higher levels of satisfaction lead to continued use of the same service? Are patients more apt to comply with treatment regimen if they are more satisfied with care? The literature generally confirms that satisfaction and utilization of health services are positively related (43). Because most studies are cross-sectional instead of longitudinal, it is not easy to establish the direction of causality; for instance, does higher satisfaction result in more utilization or is it the other way around? Findings consistently show that dissatisfaction is linked to the intention to switch services or to reports of terminating services (43).

On the factors associated with patient satisfaction, the χ^2^ values obtained showed that at *P* < .05, there was a statistically significant association between level of satisfaction and patient’s marital status and the highest level of educational attainment. Most of the participants, who were married (98.2%), were satisfied with services followed by those who were single and then those who were divorced or widowed. Also, a higher percentage of respondents with lower levels of education reported being satisfied, whereas the highest percentage of those who were dissatisfied were respondents with postgraduate degrees. This may be so because people with higher levels of education oftentimes may have a prior knowledge of how they ought to be treated and so have higher expectations of the quality of service received than their counterparts with lower education levels. On the other hand, no statistically significant association was found with age, gender, ethnicity, employment status, years of attending physiotherapy clinic, and medical diagnosis of respondents. This contrasts with the results of a study that assessed the service quality of physiotherapy services in a teaching hospital in Klang Valley, Malaysia, which found significant associations between patient satisfaction and gender, ethnic group, marital status, and waiting time (46,47). Other studies have also found age as the most consistent factor associated with patient satisfaction with older patients more satisfied with care (47,48). Inconsistent results were found with education, gender, social class, income, marital status, and race (47,48). In a meta-analysis of 221 studies, the results found greater satisfaction with being older, having less education, having a higher social status, and being married (49) One study reported that satisfaction with health outcomes did not differ with patient’s age; however, patients aged 65 years and older were more satisfied with access to health services and effectiveness of communication (50). Higher proportions of women than men have also reported complete satisfaction with care (49). Differences in satisfaction between male and female patients were identified in a study; for the male patients, the main predictors of satisfaction were the therapist and treatment outcome, whereas for female patients, the main predictors were organization and communication (51). The expectation of care dimension of satisfaction was significantly higher in male patients than in female patients (51).

There was a statistically significant association between satisfaction with the cost of service received and overall satisfaction with physiotherapy services among the respondents. A high percentage of respondents who were satisfied with the cost of service were also satisfied with the overall physiotherapy services received, with a high percentage of those dissatisfied with cost also showing dissatisfaction with overall physiotherapy services. This result points to the importance of the cost of care to patients. It is worthy of note that patients who felt that the cost was high or unaffordable were not satisfied with the services received and as such may not use the service again or recommend it to other people. This finding agrees with existing studies which reported that patients with health insurance had a higher satisfaction level with health-care professional services than those paying out of pocket (52,53). While the findings in our study agrees with the existing literature, this may not be the case if most patients who use the teaching hospitals in Nigeria do not pay out of pocket. More so, most times, physiotherapy care is typically long in the developing context like Nigeria, often with no discharge period, and so it is understandable if the cost of care influences a patient’s satisfaction with services.

No significant association was found between responses about waiting time and overall satisfaction with physiotherapy services, although a large body of literature (2,32,37,39,54,55) had mentioned long waiting time as one of the major determinants of satisfaction.

In addition, this study acknowledges that some other factors like the physiotherapist’s attributes such as ethnicity, affiliations, duration of practice, and professional behaviors (eg, communication patterns and level of professional expertise) may affect patients’ satisfaction. Also, constant changing of physiotherapists between sessions or/and at subsequent visits may influence how patients rate their satisfaction to physiotherapy care received. While some other factors may exist, it was difficult to capture these factors in the questionnaire used in this study. Therefore, there is a need to conceptually develop a questionnaire that would explore more in-depth the factors that affect patient’s satisfaction to care. An explorative qualitative study to understand patients-centered factors to care would be a starting point.

### Study Limitations

Since the patients have been attending the clinic for at least 6 months, there is a possibility of recall error, especially with waiting time. More so, collecting information on the characteristics of the physiotherapists (eg, age, gender, and years of experience of the physiotherapist) attending to the participants in this study would have provided an insight into whether these attributes can influence the patient’s satisfaction level. Furthermore, the absence of a qualitative component to further explore in detail the various factors that made the satisfaction high is a limitation to this study. Therefore, a mixed method approach would have been appropriate in understanding the patients’ satisfaction in physiotherapy clinics.

The generalization of these findings should be applied with caution, since the findings are specific to tertiary hospitals and to a particular region of Nigeria. Nevertheless, our study has contributed immensely to the literature by conducting some psychometric properties of the PTPSQ in Nigerian patient population attending 2 outpatient physical therapy clinics in Lagos. However, we failed to conduct certain tests such as construct validity (eg, convergent validity) to ascertain if there is a high correlation between the modified PTPSQ and the original PTPSQ. Although not the aim of this study, we believed that conducting analysis to see if there are differences between the 4 domains that measure satisfaction and which of the domains is most influenced by the demographic factors would have been a great contribution to the tool used in this study.

We conclude that patients attending physiotherapy services in the tertiary hospitals in Lagos, Nigeria, were satisfied or very satisfied in the overall domain that measures satisfaction and 2 other domains: physiotherapy services and staff attitude. However, they were more indifferent to some of the items in the domain that measure the level of satisfaction with physiotherapy accessibility and location. Therefore, we recommend that in designing physiotherapy clinics, the location as well as the features of accessibility as defined by the World Health Organization should be considered.

## Appendix A

### Questionnaire

#### Demographics

1. **Age** -------------
2. **Sex:** Male…………. Female………
3. **Marital Status:** Single……. Married……. Divorced…………Widowed………. Others………
4. **Educational Status:** Never attended any school…………Primary………. Secondary……. Diploma……………BSc………. MSc/PhD……………
5. **Occupational Status:** Private Service………. Government service…. Self-employed………. Student………. Others……….
6. **Insurance Status:** Insured………………. Uninsured………….
7. **Insurance Type:** NHIS………………… Voluntary…………. Community based health insurance…………………, Others, please specify……………
8. **Ethnicity:** Yoruba………. Igbo………… Hausa………. Others, please specify…….
9. **Clinic Unit Visited:** Orthopedics…………. Neurology…………………. Obstetrics & Gynecology……………. General Health Promotion………………. Others, please specify………. **(9b)** How long (years/months) have you being attending this clinic……….
10. **The patients self-identified diagnosis for visiting the clinic:……………**

### Patient Satisfaction Scale

Please rate your degree of satisfaction with each of the following statements. 1 = very dissatisfied, 2 = dissatisfied, 3 = indifferent, 4 = satisfied, 5 =very satisfied.

**Table:**

S/N	Physiotherapy accessibility (n = 5)	1	2	3	4	5
1	The location of the hospital was convenient for me					
2	Parking was available for me					
3	It was easy to get the physiotherapy clinic					
4	The distance to get to the facility is acceptable to me					
5	It was an easy admission and administrative procedure					
	Physiotherapy services (n = 6)					
6	I was given privacy when I need it					
7	The clinic scheduled appointments at convenient times					
8	My first visit for physiotherapy was scheduled quickly					
9	It was easy to schedule visits after my first appointment					
10	The waiting time was short					
11	The physiotherapy rooms were calm and relaxing					
	Staff attitude (n = 6)					
12	The non-clinical staff were helpful and courteous					
13	The physiotherapist was courteous					
14	My physiotherapist understood my problems or conditions					
15	My physiotherapist put me at ease and reassured me					
16	My physiotherapist explained the treatment process to me					
17	The instructions my physiotherapist gave me was helpful					
	Overall satisfaction (n = 6)					
18	The quality of care I receive is equivalent with the cost					
19	The cost of the therapy received was reasonable					
20	I would recommend this physiotherapy clinic to family and friends					
21	I would return to this physiotherapy clinic if I required therapy in future					
22	I was satisfied with the overall quality of my therapy					
23	Overall, I was satisfied with my physiotherapy experience					
